# Experiences in supporting the structured collection of cancer nanotechnology data using caNanoLab

**DOI:** 10.3762/bjnano.6.161

**Published:** 2015-07-21

**Authors:** Stephanie A Morris, Sharon Gaheen, Michal Lijowski, Mervi Heiskanen, Juli Klemm

**Affiliations:** 1 Office of Cancer Nanotechnology Research, National Cancer Institute/NIH, 31 Center Drive, Bethesda, MD, 20892, USA; 2 Leidos Biomedical Research, Inc., Frederick National Laboratory for Cancer Research, 8560 Progress Drive, Frederick, MD, 21701, USA; 3 Essential Software, Inc., 9024 Mistwood Drive, Potomac, MD, 20854, USA; 4 Center for Biomedical Informatics and Information Technology, National Cancer Institute/NIH, 9609 Medical Center Drive, Rockville, MD, 20850, USA

**Keywords:** caNanoLab, cancer research, databases, nanomaterials, nanomedicine

## Abstract

The cancer Nanotechnology Laboratory (caNanoLab) data portal is an online nanomaterial database that allows users to submit and retrieve information on well-characterized nanomaterials, including composition, in vitro and in vivo experimental characterizations, experimental protocols, and related publications. Initiated in 2006, caNanoLab serves as an established resource with an infrastructure supporting the structured collection of nanotechnology data to address the needs of the cancer biomedical and nanotechnology communities. The portal contains over 1,000 curated nanomaterial data records that are publicly accessible for review, comparison, and re-use, with the ultimate goal of accelerating the translation of nanotechnology-based cancer therapeutics, diagnostics, and imaging agents to the clinic. In this paper, we will discuss challenges associated with developing a nanomaterial database and recognized needs for nanotechnology data curation and sharing in the biomedical research community. We will also describe the latest version of caNanoLab, caNanoLab 2.0, which includes enhancements and new features to improve usability such as personalized views of data and enhanced search and navigation.

## Introduction

The U.S. annual report to the nation on the state of cancer indicates a steady decline in overall mortality rates, with increases in incidence for many cancers [1]. Internationally, cancer incidence paints a more dramatic picture in which the number of new cases has increased from 12.7 million in 2008 to 14.1 million in 2012, with this number expected to rise even further by an additional 75% in the next two decades [2]. Regardless of whether the focus is limited to the U.S. or considered internationally, the implied and actual burden of cancer is clear, calling for earlier detection and treatment modalities to alleviate this problem. Standard cancer therapeutics are often characterized by poor water solubility and rapid degradation leading to narrow therapeutic windows and doses limited by toxicity [3]. In turn, diagnostics are often hindered at the level of sensitivity, and time between testing and diagnosis. Opportunities for the potential to improve current cancer therapeutics and diagnostics are sorely needed. Nanotechnology provides tremendous opportunities in applications to medicine to make improvements in both these areas. At the nanoscale, the properties of materials yield unique chemical, physical, and biological features that make them advantageous drug delivery vehicles and imaging agents that can target tumor cells, while sparing healthy cells – thereby drastically reducing the toxicity of treatments [4]. Even more so, nanotechnology can be utilized to deliver newer drugs that in the absence of nanotechnology-based vehicle are undeliverable at effective doses [5].

Yet, major hurdles remain to be overcome before we can expect to see regular use of nanotechnology in the clinic that are inherent to new technologies at the clinical trial stage, such as the cost of development, and biological challenges that need to be addressed to ensure patient safety and efficacy. There are only five U.S. Food and Drug administration approved nanotechnology-based drugs – Doxil, DaunoXome, DepoCyt, Marqibo, and Abraxane – while many more are in clinical trials [6]. Similarly, there are a limited number of approved diagnostic devices and tests [7]. In other areas of research, especially genomics, the sharing of experimental data has been shown to be vital for the advancement of scientific discovery and translation [8–9]. Databases such as dbGaP have provided investigators access to hundreds of genomics studies, resulting in three times that number of publications and scientific advances in the genetic basis of disease [8]. Unlike genomics, nanotechnology data management systems, which are at relatively early stages of development, must consider the heterogeneity of nanomaterial data and varied needs based on application (e.g., research focus – environmental vs medical vs energy). Even within a given research area, multi-disciplinary contributions to the field further complicate the development of management systems that address the needs of different communities.

The task of creating relevant databases for nanotechnology risk assessment, manufacturing, characterizations, and literature data is being taken on globally by government, academic, and regulatory organizations. To date, there are approximately 38 databases at various stages of development from initial schema integration to storage of structured, accessible data [10]. However, obstacles still exist in accessing well-characterized datasets and computational tools for further analyses, validation, and guidance in the design optimization of nanomaterials. Further, the development and adoption of data standards to enable efficient data deposition into databases and sharing between laboratories and individual investigators is of great importance. Building the infrastructure for organized data management systems is seen as a potential avenue to overcome these challenges to technology development and clinical translation.

Here we discuss considerations for developing a user-friendly nanomaterial repository in biomedicine and sharing well annotated nanotechnology data. In particular, we describe the cancer Nanotechnology Laboratory (caNanoLab) data portal, a web-based database that allows users to submit and retrieve information on highly described nanomaterials used in biomedicine. We provide an overview of caNanoLab functionality and the release of caNanoLab 2.0, which contains new features and enhancements that address some of the barriers to data sharing described above and enable more efficient data submission and greater support for users.

## Results and Discussion

### caNanoLab 2.0 navigation, search, and submission

**Figure 1:**
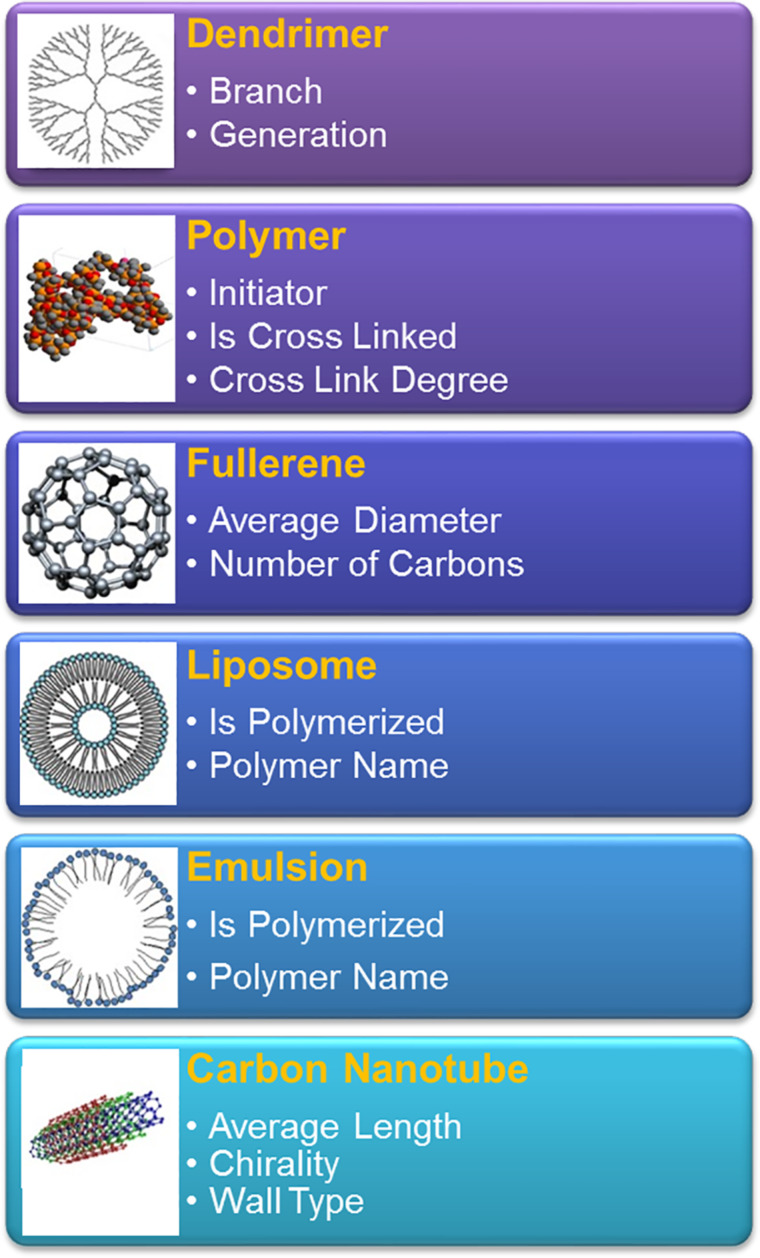
Example nanomaterial types supported by caNanoLab. Pictured is a subset of supported nanomaterials, along with a list of example high-level metadata specific for the listed particle types.

As we have previously reported, the caNanoLab project (https://cananolab.nci.nih.gov/) was initiated as a collaborative effort between the National Cancer Institute’s (NCI) Office of Cancer Nanotechnology Research and Center for Biomedical Informatics and Information Technology to address the characterization requirements for federal regulatory review of nanomaterial-based investigational new drugs, diagnostic devices, and imaging agents [11–12]. caNanoLab was originally designed to capture information about the nanomaterial sample and its composition, associated in vitro characterizations, experimental protocols, and relevant publications. The ultimate goal being to accelerate the clinical use of cancer nanomedicines by providing efficacy and safety information to support the above mentioned review process for the use of these nanomaterial in human cancer clinical trials, one of the first step to clinical use. Moreover, caNanoLab was designed to enable the sharing of highly described and complete nanomaterial datasets that can then be re-used for downstream analyses and nanomaterial optimization. In the past decade since its launch, caNanoLab has been expanded to further address the needs of the biomedical research community by enabling the submission and retrieval of diverse nanomaterial types (Figure 1) and characterizations, including in vivo and ex vivo characterizations, to additionally support computational modeling and simulation of nanoparticle behavior. Standardized metadata are provided to aid these efforts.

### caNanoLab navigation and search features

**Figure 2:**
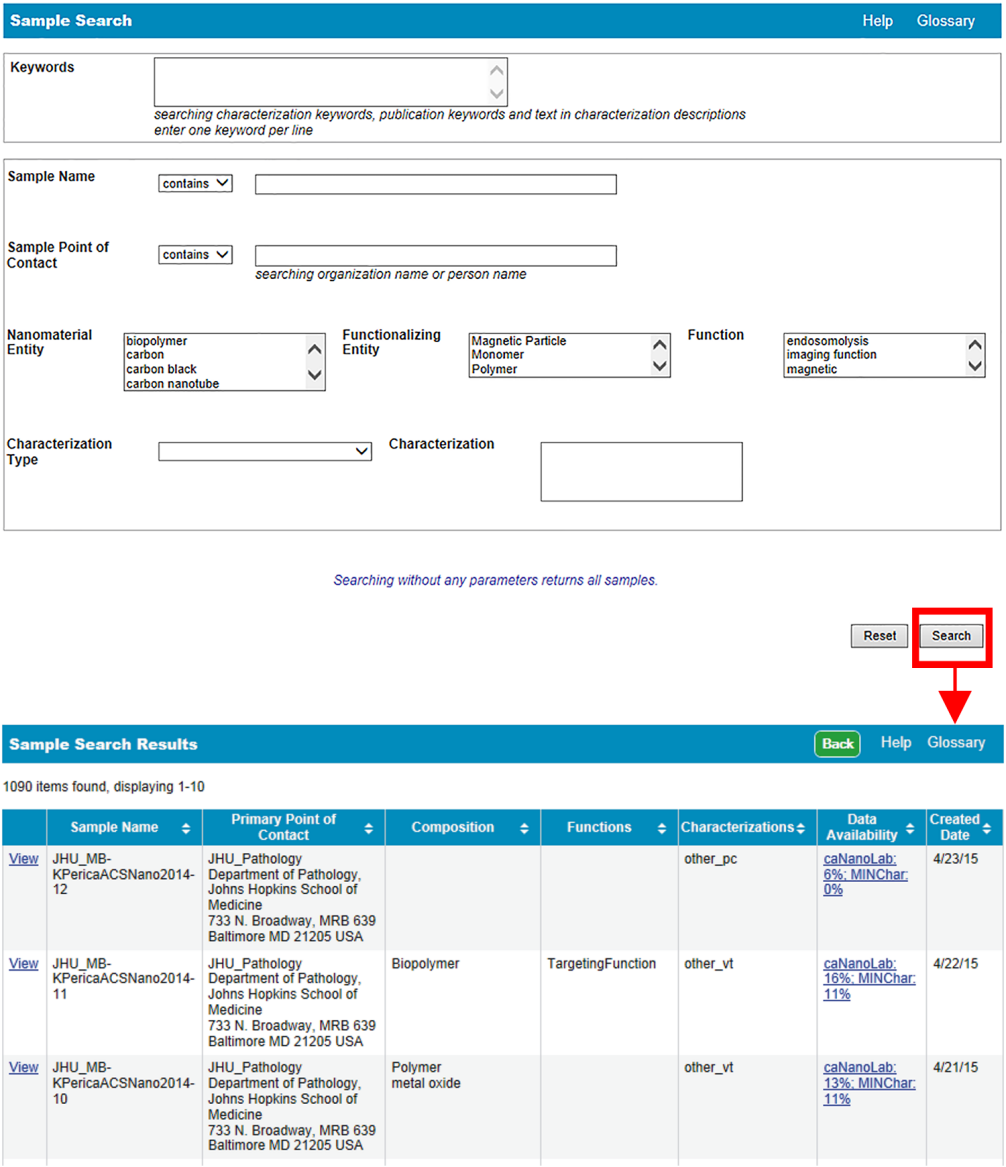
Sample search. Users can search for samples by keyword, name, point of contact, or feature. Following a search (red highlighted box and arrow), users are taken to a sample search results screen from which users can review the results and select sample records to view.

In support of data sharing, caNanoLab compliments other nanomaterial data resources [11] and provides facilities that enable the retrieval and submission of standardized nanomaterial data. Currently, more than 1,000 curated nanomaterial records are publicly accessible and can be queried directly from the caNanoLab homepage. Web usage statistics indicate the majority of users are from the U.S., but has grown to include users from several other countries such as Great Britain, Germany, China, the Netherlands, Spain, and Japan. In 2014, the number of unique portal visitors numbered over 3,000. Options for browsing curated protocols, samples, and publications are available on the homepage. In the caNanoLab 2.0 release, the homepage layout and interface were changed to improve navigation, including enhancements to the User Actions options, and access to commonly asked questions and answers. By selecting “Search Samples,” users are taken to a screen from which nanomaterial samples can be queried by keyword, name, or nanomaterial feature. Each sample provides information on the nanomaterial developer, which is also provided as a search option (Sample Point of Contact), and listed in detail in the subsequent Sample Search Results screen (Figure 2).

**Figure 3:**
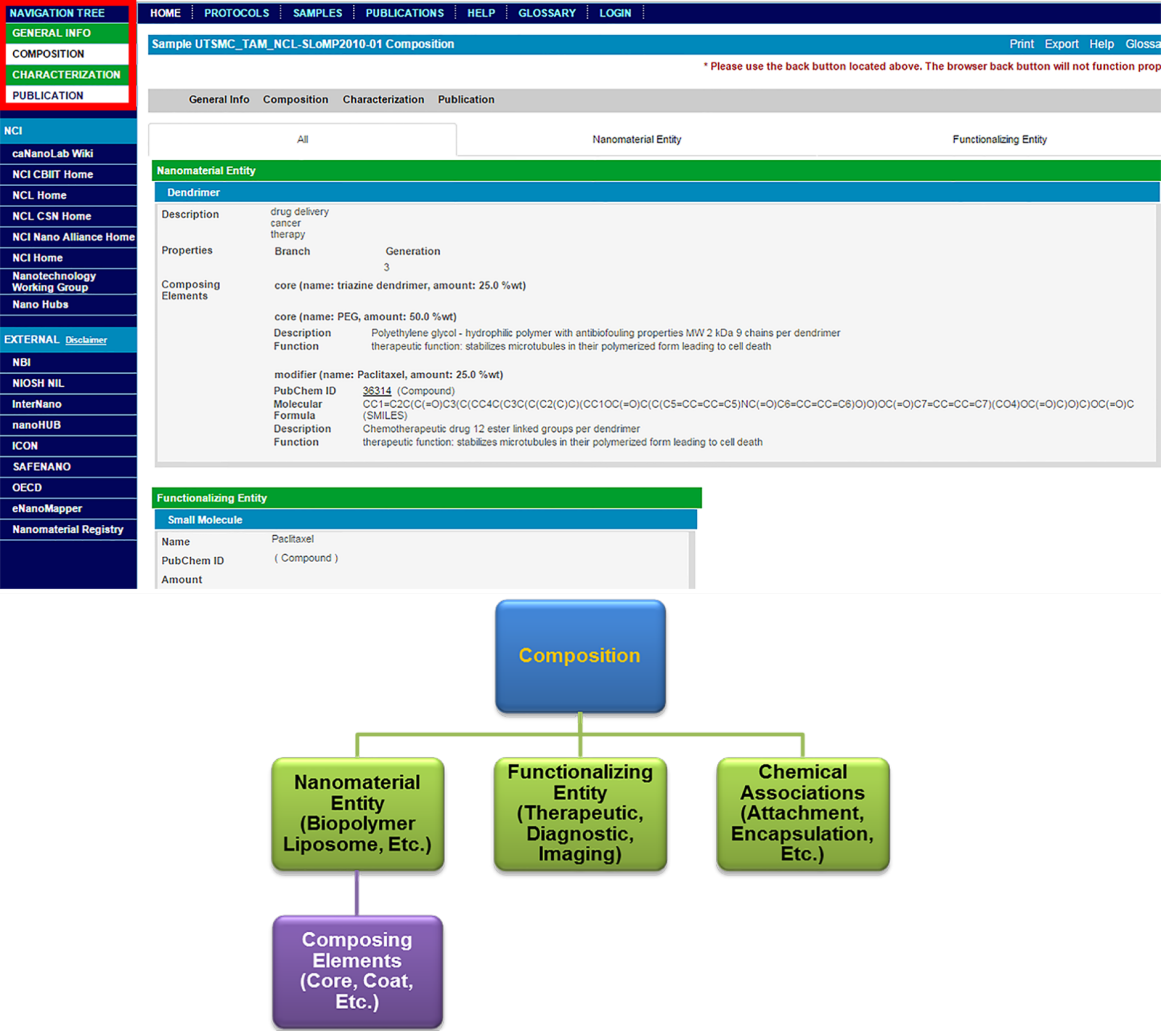
Example nanoparticle composition in caNanoLab of a triazine dendrimer with paclitaxel. Composition information captures properties inherent to the dendrimer (e.g., generation), as well as properties inherent to several particle types (e.g., chemical name, molecular formula). Bottom diagram highlights high level concepts and properties pertaining to composition.

**Figure 4:**
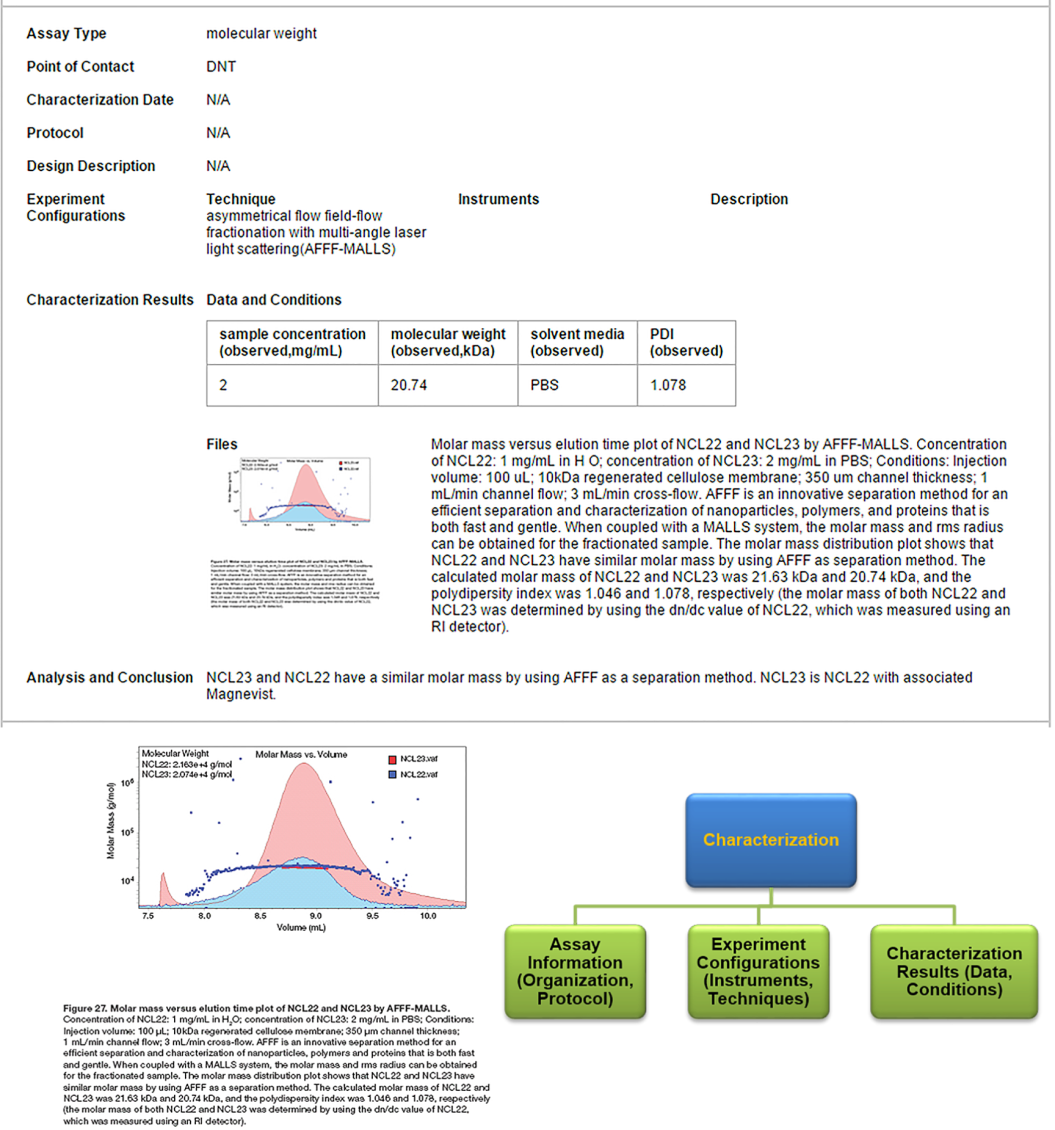
Example Nanoparticle Characterization in caNanoLab of a Dendrimer. Characterization information captures information about the assay type and experimental conditions (e.g., technique, concentrations, and observed measurements). Pictured here is an example in which the molecular weights of two curated nanomaterials are compared by light scattering (top and bottom left). Bottom right diagram highlights parameters and factors specific to characterization assays.

By selecting “View” next to the sample of interest, users can analyze information about individual nanomaterial sample records such as composition, which includes standard metadata used to describe composition properties (Figure 3). Importantly, the “Navigation Tree” allows for viewing of other pertinent features of the selected nanomaterial such as general information about the developer (e.g., organization and role) and performed characterizations. Similarly, recommended metadata are provided for various characterization assay information such as assay type, experimental techniques, protocols, instruments, and experimental conditions to ultimately support comparison between nanomaterial studies (Figure 4). These metadata were derived from review of nanomaterial properties provided by NCI’s Nanotechnology Characterization Laboratory (http://ncl.cancer.gov/), collaborations with the NanoParticle Ontology (NPO; http://www.nano-ontology.org/), and discussions with the research community.

**Figure 5:**
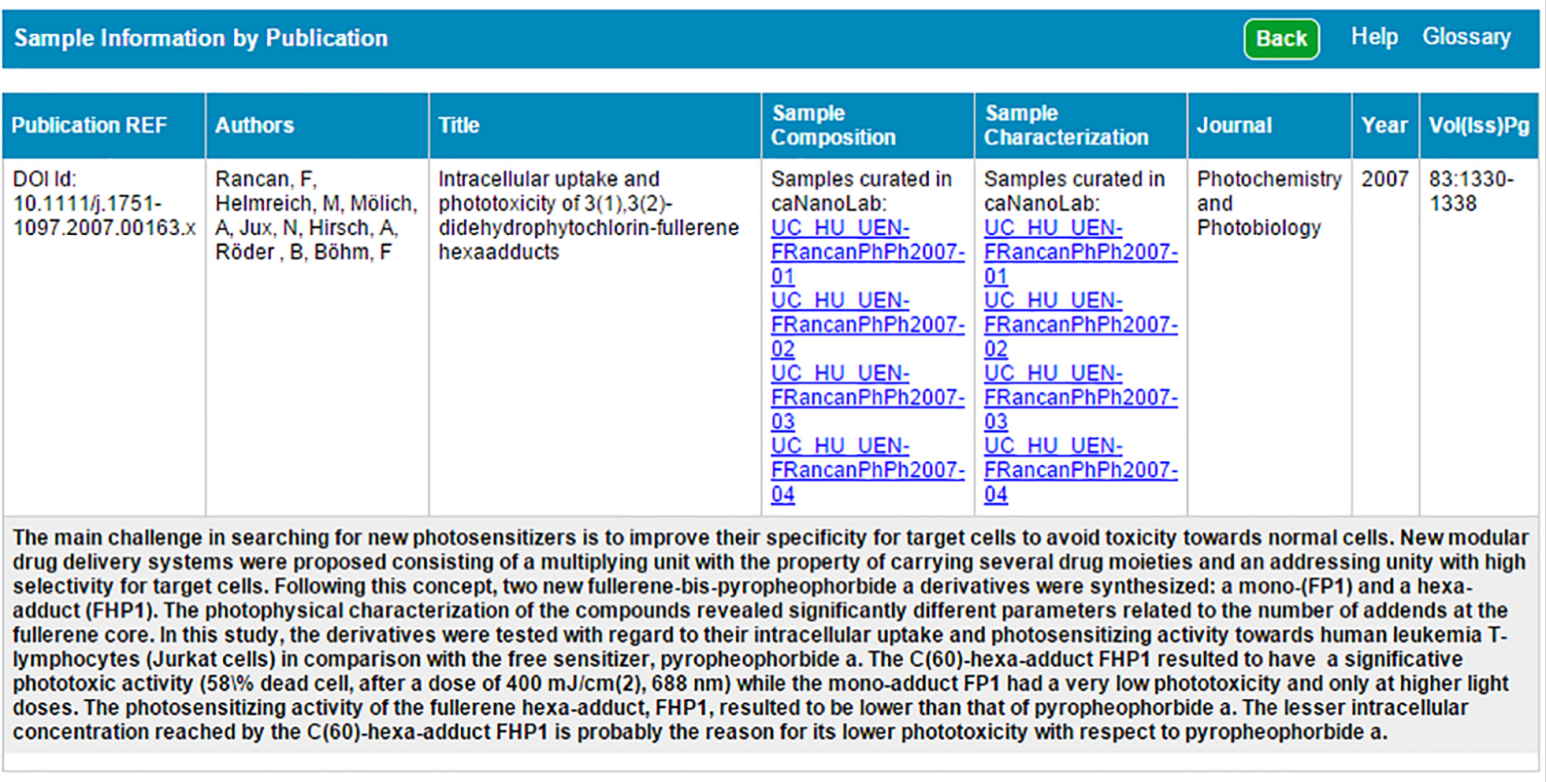
caNanoLab sample information by publication. List and active links to all curated data for a given publication are provided following a DOI-based search for samples by publication. This option is available under the Publication tab once the user has initiated a publication search.

In addition to sample searches, caNanoLab users can search for protocol and publication information by name or nanomaterial feature from the caNanoLab homepage or by using tabs at the top of a viewed nanomaterial sample record (Figure 3). Query results can be either printed or exported into spread-sheet based reports using options available on the results screen. In caNanoLab 2.0, a search for sample characterization and composition information using the associated publication’s identifier has been implemented and returns a compiled sample information page (Figure 5). Users can search by either Digital Object Identifier (DOI) or PubMed ID. This feature is also available for publication vendors to interface online articles with corresponding caNanoLab data by leveraging the publication’s DOI. By creating this interface, we hope to promote the discoverability and usage of data in caNanoLab.

### caNanoLab submission

**Figure 6:**
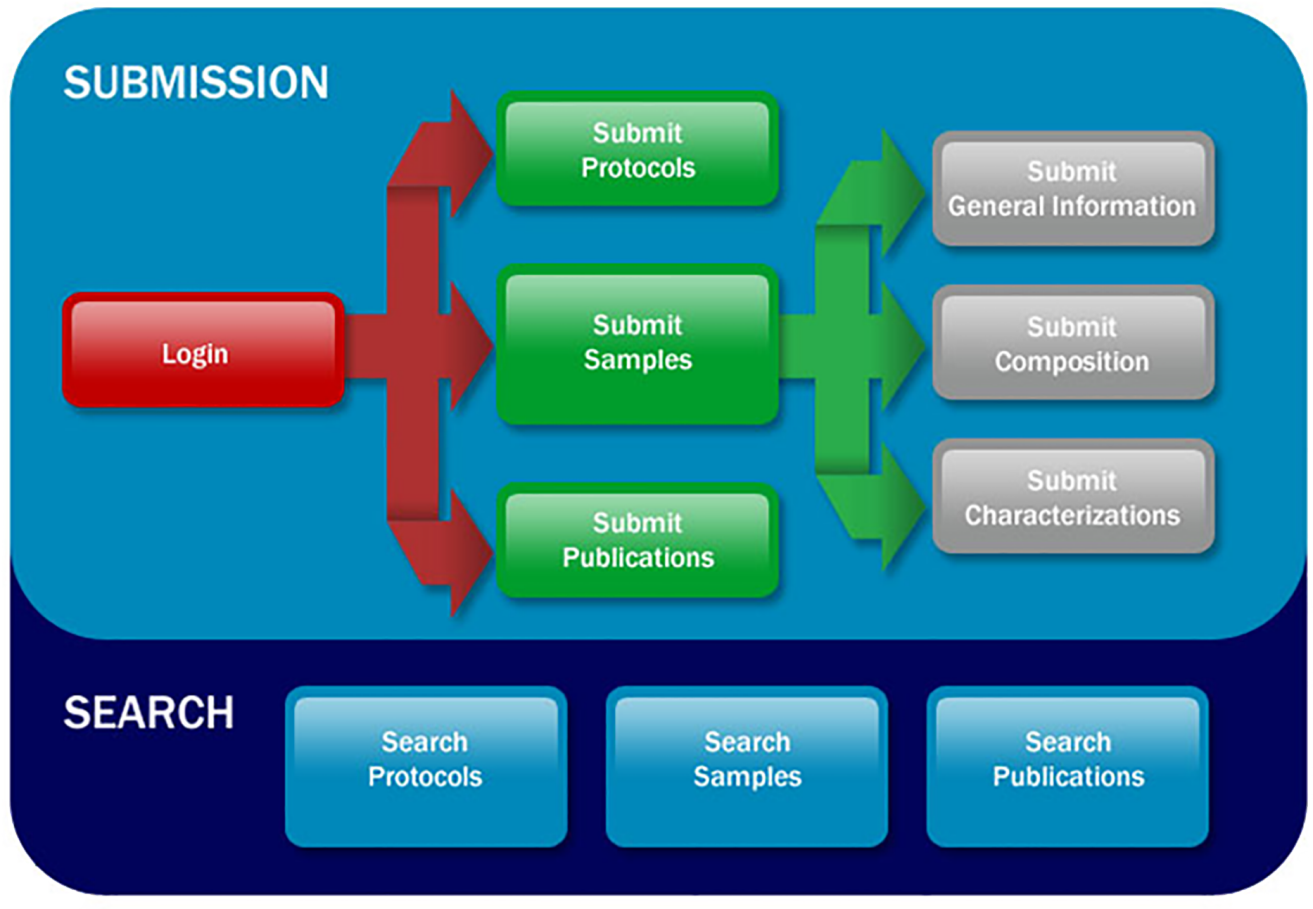
caNanoLab data submission and search workflow. A graphic available upon login that illustrates the functionality in caNanoLab. Both workflows provide active links for the indicated options. Reprinted with permission from [12]. Copyright 2014 IEEE.

**Figure 7:**
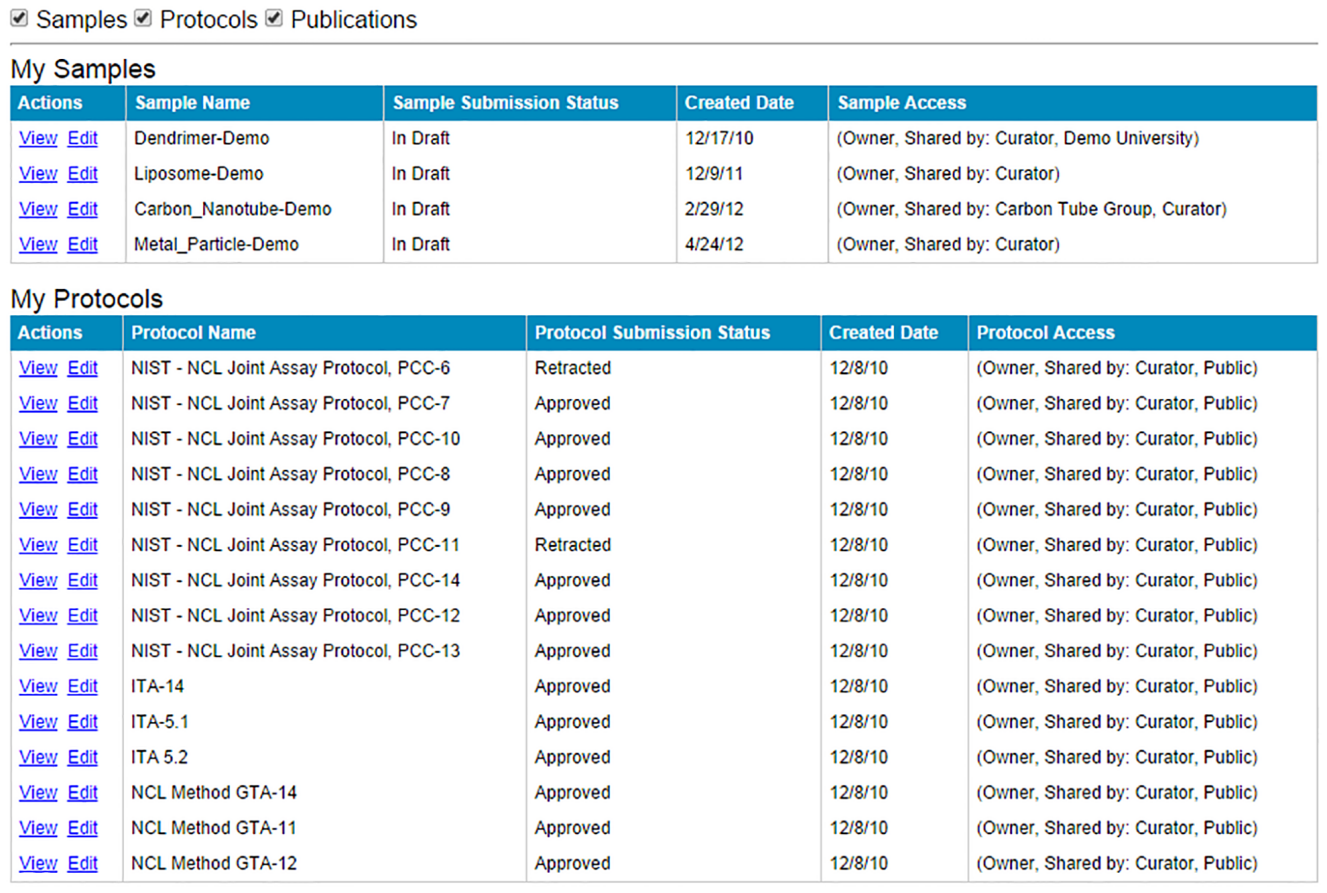
caNanoLab MyWorkspace screen.

To submit information into caNanoLab, data submitters are guided through the process with the help of a workflow diagram containing active links (Figure 6) that directs users to web-based forms. Users request an account on the homepage and once credentials are provided, may login to submit protocols, samples, and publications. All data submissions are reviewed for completeness by an in-house curator, and require approval before being made publicly available on the caNanoLab website. To improve this process, caNanoLab 2.0 introduces a MyWorkspace feature as illustrated in Figure 7 to allow submitters to view and access their submitted data, and monitor submission status.

**Figure 8:**
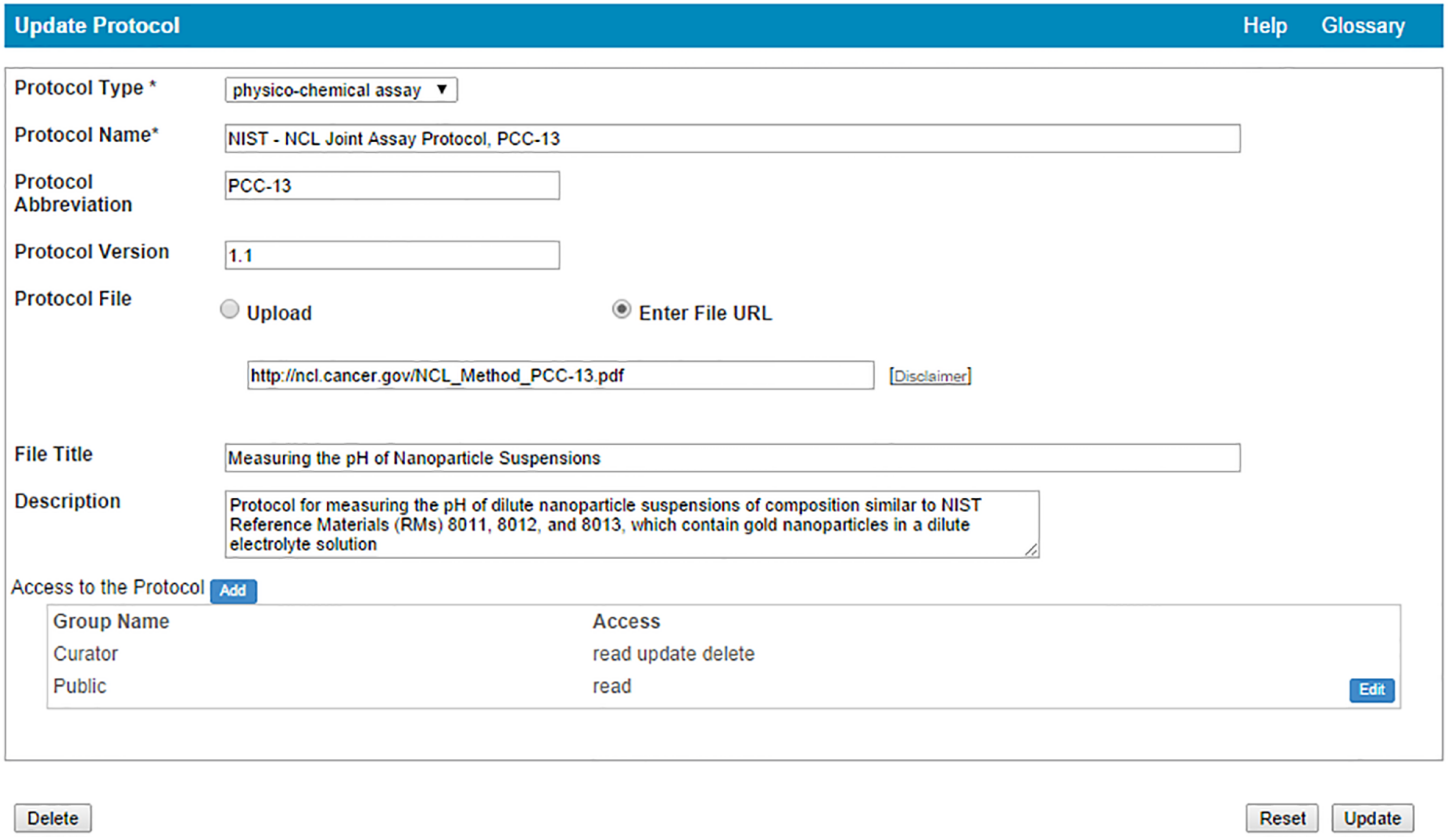
caNanoLab protocol submission screen.

Nanotechnology protocols (Figure 8) for characterization, safety, radiolabeling, sample preparation, and other detailed procedures that might be part of an experiment can be entered into the portal. Protocols currently available are primarily for physico-chemical and in vitro characterizations, however, other protocol assays are strongly encouraged and welcomed, including video-recorded procedures. Submitters can specify protocol type from a drop-down list (e.g., in vitro assay, sample preparation, other) and protocol version if multiple variations or updates exist. Protocols can be submitted as files or URLs to videos or other protocol documents maintained externally. Once submitted, protocols can then be associated with characterization assays described for submitted samples.

**Figure 9:**
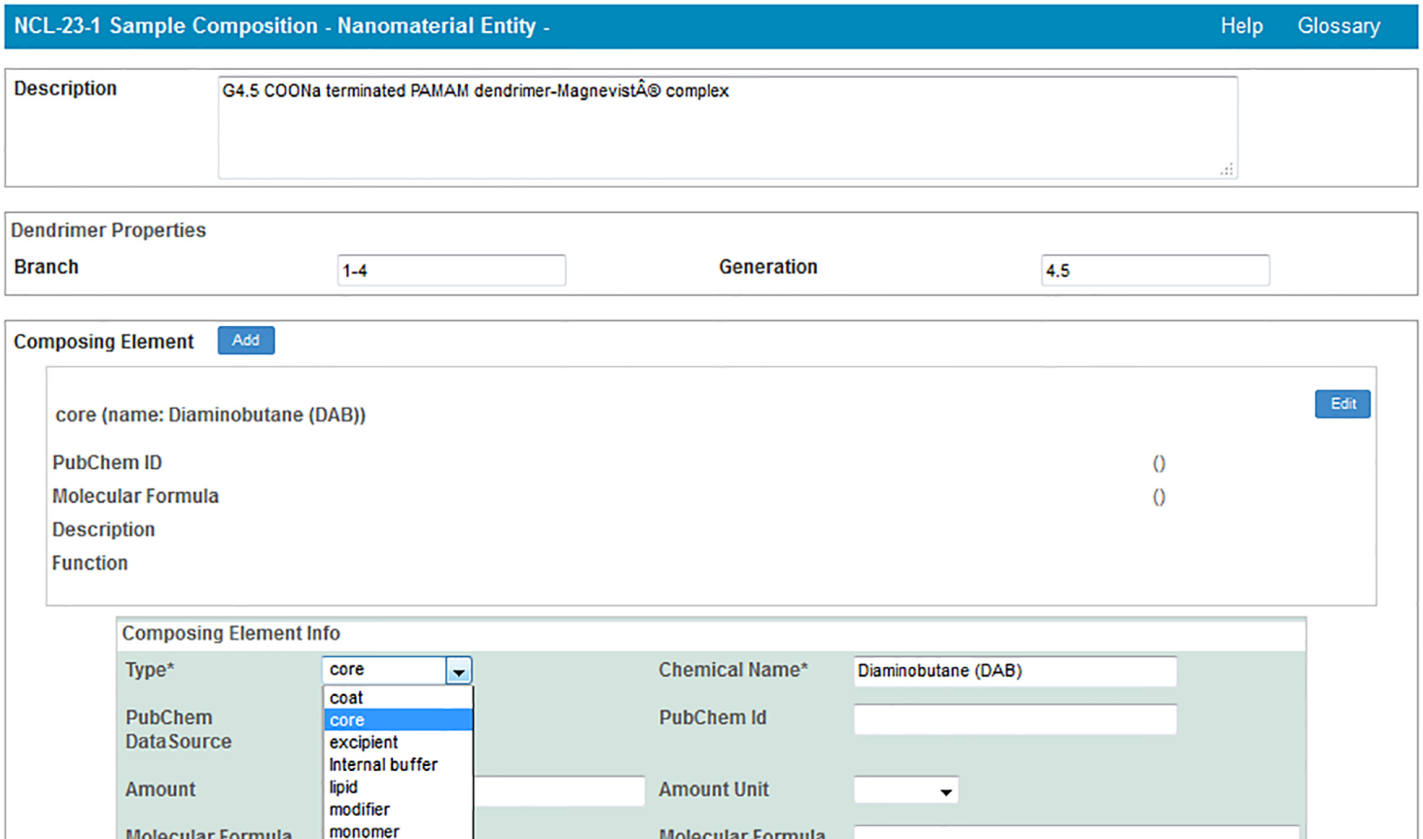
caNanoLab sample composition submission screen.

**Figure 10:**
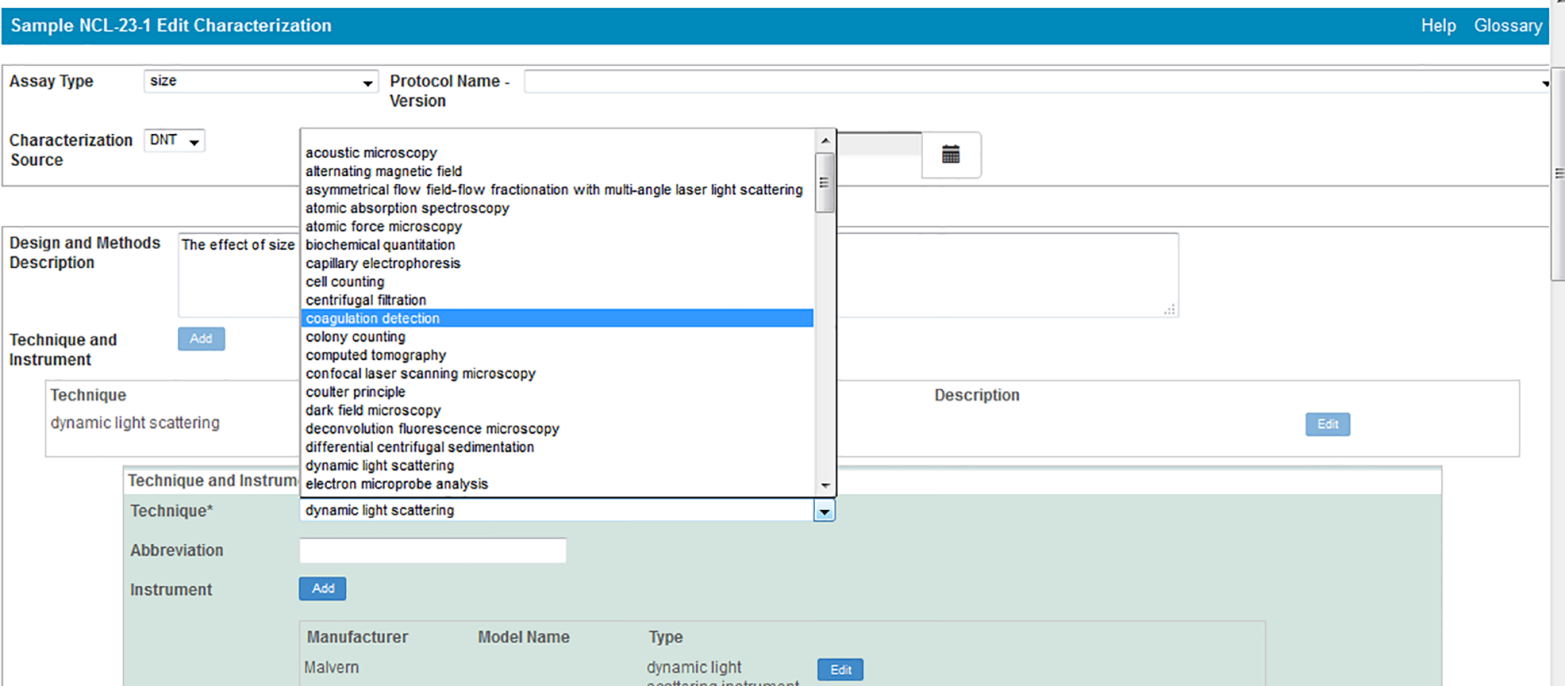
caNanoLab sample characterization submission screen – techniques and instruments.

**Figure 11:**
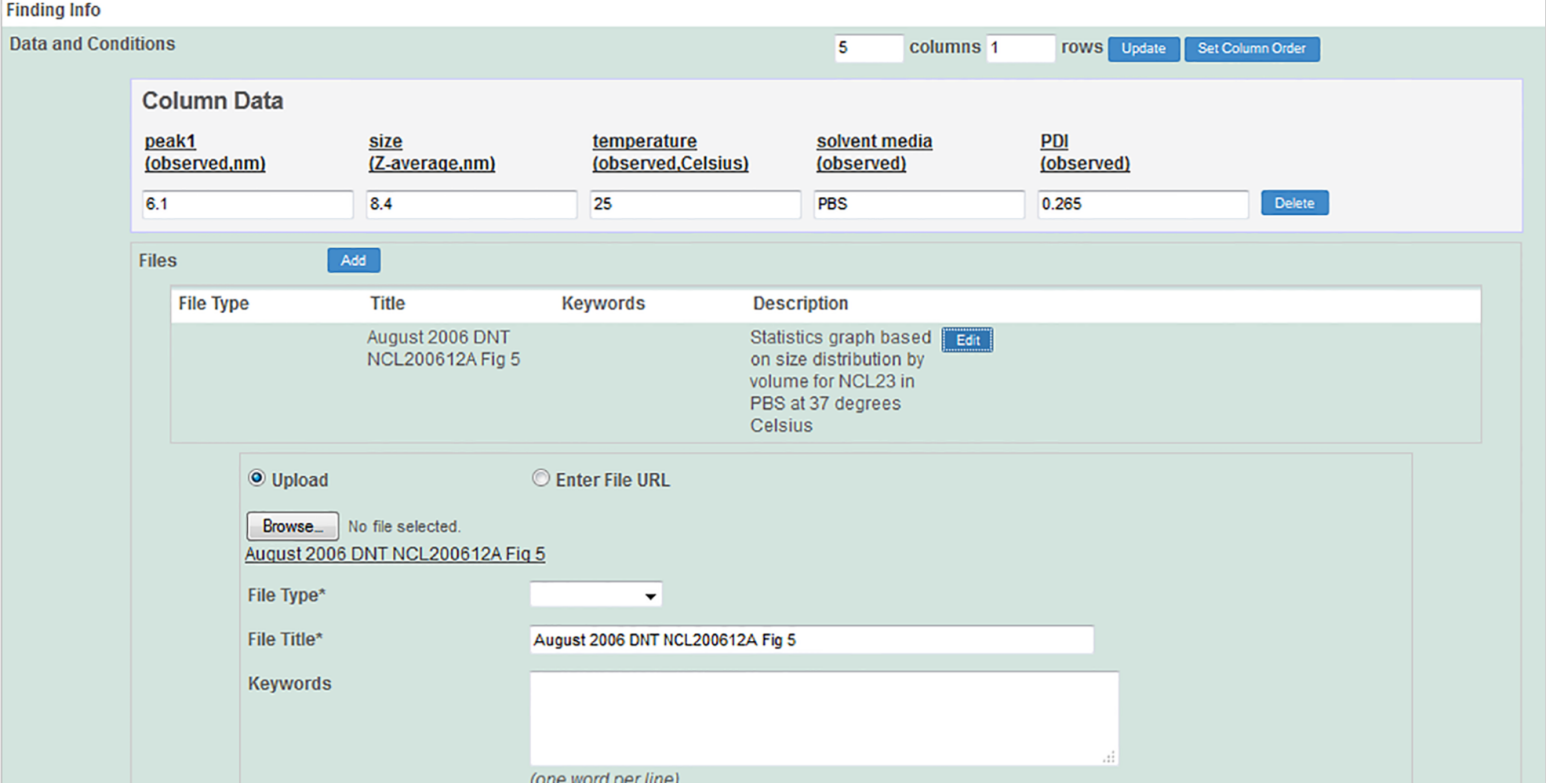
caNanoLab sample characterization submission screen – data and conditions.

**Figure 12:**
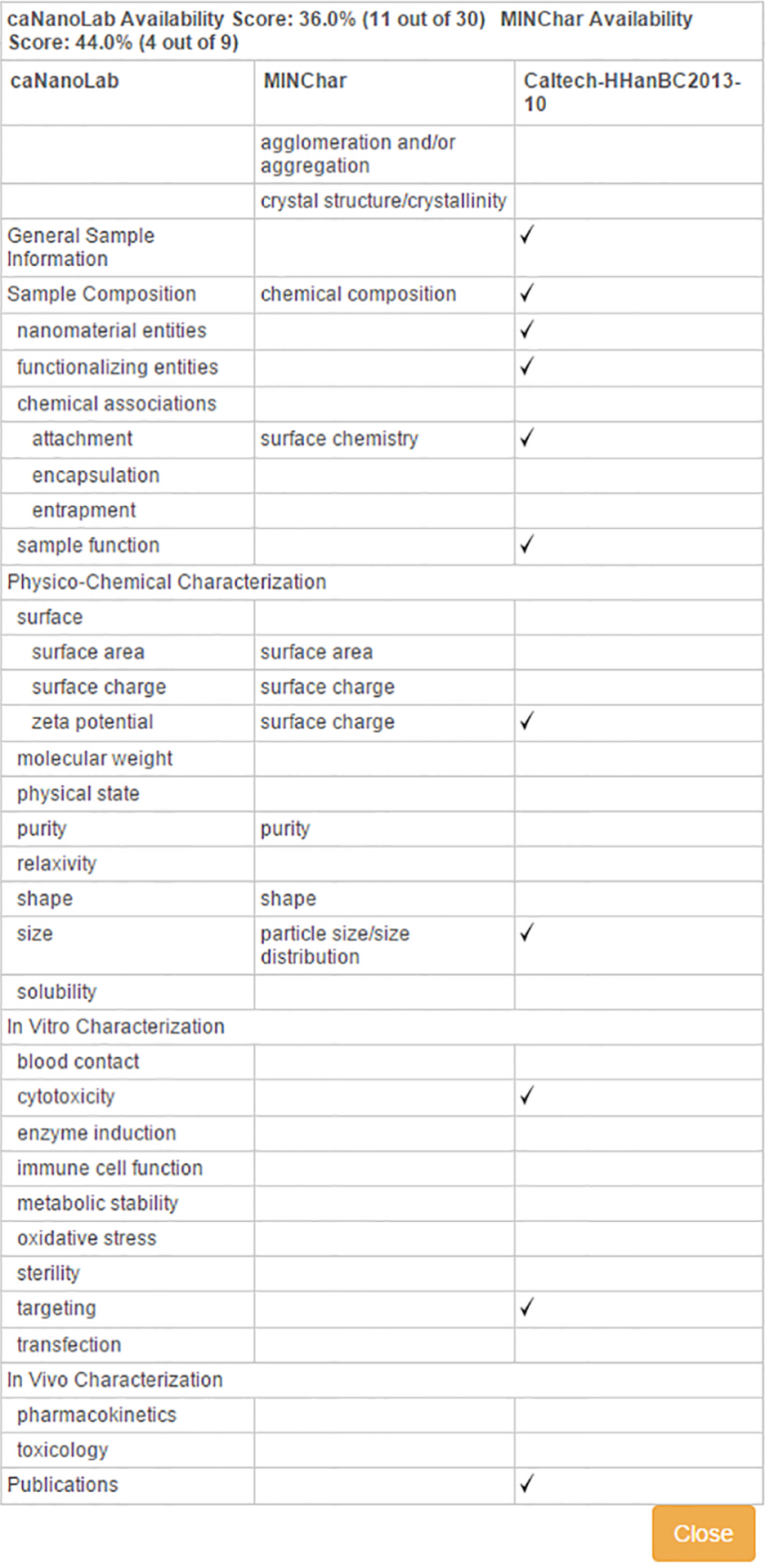
caNanoLab data availability metrics table. The first and middle columns list data supported and recommended by caNanoLab and the MinChar standard, respectively. The last column is a comparison of the data curated for the indicated sample to the caNanoLab and MinChar column lists. Data availability is provided for samples in Sample Search Results.

In addition to protocols, caNanoLab supports the submission of sample composition and characterizations. For the purposes of caNanoLab, a sample is defined as a formulation of a base nanomaterial platform and any additional components that contribute to the function(s) of the nanomaterial. Submitters can enter nanomaterial composition information (Figure 9) including: nanomaterial entities (e.g., dendrimer), functionalizing entities (e.g., small molecule), and chemical associations (e.g., covalent bond). This composition model supports the submission of complex particles (e.g., liposome encapsulated in a quantum dot) and supports the capture of properties unique to each particle type. Nanomaterial characterizations include physico-chemical, in vitro, and in vivo characterizations. When submitting characterizations, submitters can specify the protocol, instruments, and techniques used in the described characterization assay (Figure 10). Research findings information, including empirical data and experimental conditions, may also be uploaded as files and/or in a data matrix (Figure 11). Once a sample is successfully submitted to the database, either the submitter or curator can generate a data availability metrics table for the sample (Figure 12). Such a data availability metrics compares the submitted data to a checklist of data supported by caNanoLab and data recommended in the MinChar standard (https://characterizationmatters.wordpress.com/parameters/). The caNanoLab identified metadata illustrates information pertinent for nanomaterial composition and specific characterizations, while MinChar is suggested minimum metadata proposed by researchers and others involved in assessing nanomaterial safety to enable cross-comparison of nanomaterial data and data interpretation. Access to this table is available following a sample search on the sample search results screen (Figure 2).

**Figure 13:**
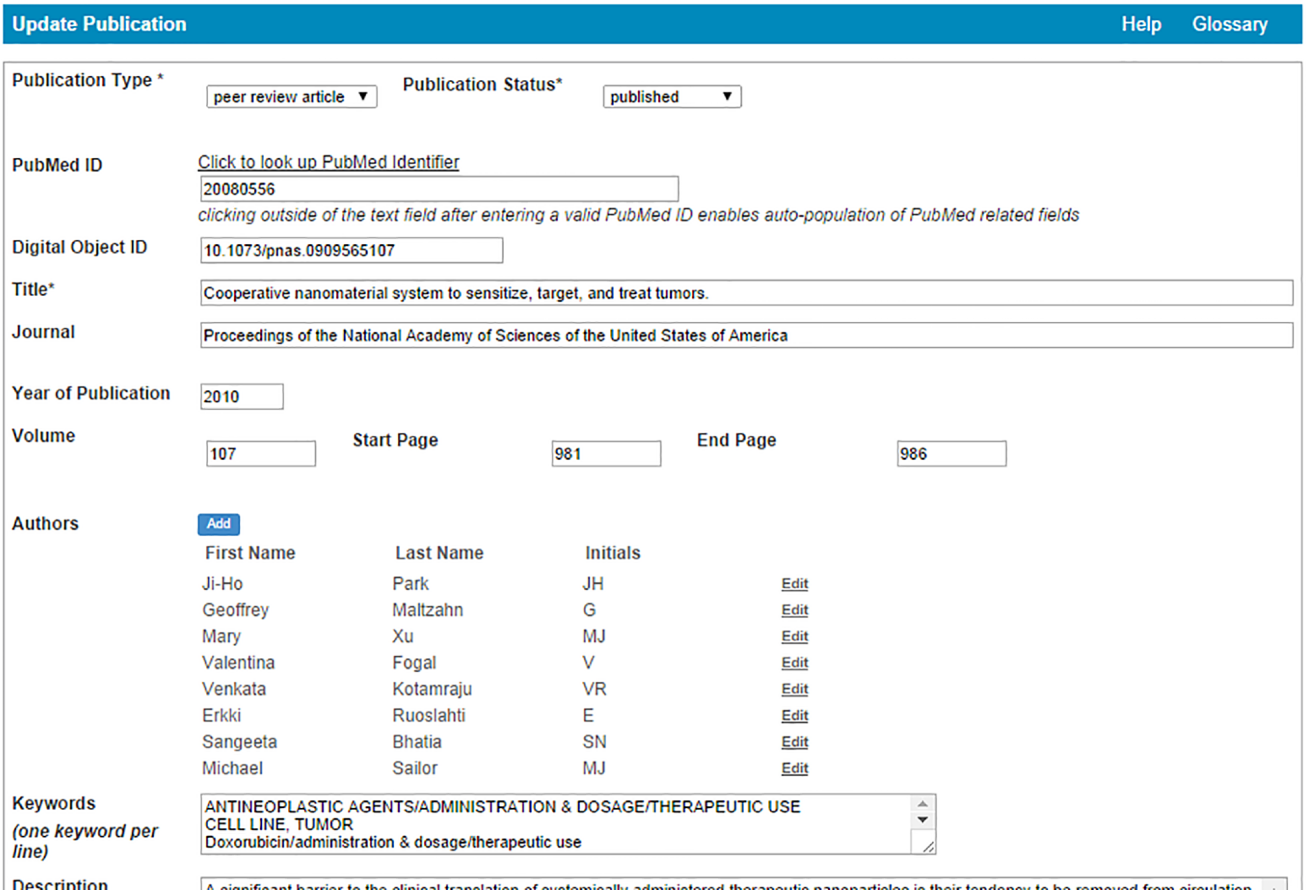
caNanoLab sample publication submission screen. Information for PubMed articles is auto-populated by leveraging PubMed’s Application Programming Interface for information retrieval.

caNanoLab also supports the submission of publications (Figure 13) and other reports. Through integration with PubMed, information about publications can be populated into caNanoLab simply by providing the PubMed ID. Previously submitted samples can be associated with a publication during the publication submission process (if samples were described in a published work), enabling the simultaneous retrieval of publication and sample information following a query.

Data submitters are allowed to make their data public or private, with the option to grant access to a limited number of users for varied levels of sharing. Submission instructions are provided in caNanoLab’s online user manual, as well as through a video tutorial that guides users through the caNanoLab 2.0 submission procedures. Both resources can be found on the caNanoLab FAQ webpage (https://wiki.nci.nih.gov/x/UKml), accessible through the caNanoLab homepage under the “How To” box. Assistance is also provided by the in-house curator.

### Data integration and sharing

To optimize the design and utility of nanomaterials in biomedicine, researchers need to integrate and compare datasets generated by different research groups. However, the lack of availability and access to datasets stored across a variety of resources with limited data exchange hinders this goal. The caNanoLab team strongly supports interoperability between databases, and engages in activities focused on the development of standards to enable data exchange. In particular, the design of the caNanoLab data model was informed by the NPO, which represents knowledge underlying the description, preparation, and characterization of nanomaterials in cancer nanotechnology research [13]. caNanoLab data model class names and attributes are maintained in the NCI cancer Data Standards Repository (https://cdebrowser.nci.nih.gov/CDEBrowser/), and definitions for caNanoLab concepts are maintained in the NCI Thesaurus (http://ncit.nci.nih.gov/). The caNanoLab team is also working with the ISA-TAB (http://isatab.sourceforge.net/) and nanotechnology communities to develop a specification that provides descriptive information applicable to nanotechnology using spreadsheet-based file formats – ISA-TAB-Nano [14]. Curated caNanoLab data are annotated by terms from Bioportal (http://bioportal.bioontology.org) and entered into ISA-TAB-Nano files that are available for download at https://wiki.nci.nih.gov/x/lgFwBg by individual users or other databases to enable data exchange.

In addition to the development and utilization of data exchange standards, another challenge to data sharing, as viewed by caNanoLab, has been access to investigator-derived data, and submission of these data by individual investigators. The majority of data submitted into caNanoLab are curated from published articles. The most challenging aspect of this process is acquiring additional information from the author. To address this challenge, many of the features in caNanoLab 2.0 to enhance navigation and enable personalized views of data were designed to improve individual investigator/user data submission. Further, the NCI Alliance for Nanotechnology in Cancer program (http://nano.cancer.gov), a network of extramural research centers and projects also supported by NCI’s Office of Cancer Nanotechnology Research, now requires awardees to share data through appropriate publicly accessible databases such as caNanoLab, and has made nanomaterial data deposition a Term and Condition of award (see RFA-CA-14-013 (http://grants.nih.gov/grants/guide/rfa-files/RFA-CA-14-013.html); PAR-14-25 (http://grants.nih.gov/grants/guide/pa-files/PAR-14-285.html)). A nanomaterial data sharing coordinator must be named for each award and plans for data sharing must be included with each application submission. Information on how to incorporate the use of caNanoLab into a data sharing plan is available on the caNanoLab website to make this process easier. Although this is not yet a requirement for other nanomaterial-related funding opportunity announcements, NCI’s Office of Cancer Nanotechnology Research hopes this will encourage data sharing and acceptance of nanomaterial data deposition as a standard practice similar to what has been observed for genomics data and currently instituted federal data sharing policies [8,15].

### Addressing future needs of biomedical databases supporting nanotechnology

The genomics community expressed the need for standards and databases to house the extensive amount of data generated by gene expression and sequencing experiments, yielding such efforts as the development of the minimum information about a microarray experiment (MIAME) [16]. As a result, the MIAME guideline, and others, have been adopted by journals, databases, and researchers as an accepted format for annotating data – a requirement called for by these groups [17]. Similarly, in order for the nanoinformatics field to grow, the relevance of nanotechnology data and associated information must be emphasized by the community. In discussions amongst community members, primarily in consultation with journals, researchers acknowledged and agreed with the importance of implementing minimum characterization requirements and guidelines, but the manner in which to identify these features were debated [18]. Different types of information are needed based on the purpose of the study, which may vary based on the nanotechnology application [19]. Considering these issues, caNanoLab and other nanomaterial databases require input and support from users including informatics experts, nanotechnologists, biologists, and clinicians to better understand their needs. Active outreach and collaborations are required to meet these goals, as well as sustained interest in the use of databases by the community, and increased data exchange between resources and researchers.

### Enhancing data interoperability by collaborative development of data standards and best practices

The caNanoLab team is engaged in many activities to better serve the needs of the nanotechnology research community and increase adoption of caNanoLab and other nanomaterial resources. Activities range from engaging publication vendors to facilitate linkages between publications and nanotechnology databases (as described above), to working with other groups to develop data standards and guidelines for data submission and sharing. In particular, interoperability with other databases is seen as important both for NCI and the caNanoLab user community. To achieve this goal, the caNanoLab team actively works with other databases, community-based programs, and federal initiatives such as the National Cancer Informatics Program (NCIP) Nanotechnology Working Group (Nano WG) and the National Nanotechnology Initiative (NNI; http://www.nano.gov), to develop data standards and deposition guidelines. Accelerating the meaningful exchange of information across the nanotechnology community is a priority for the Nano WG. Consisting of researchers from academia, government, and industry, much of the group’s focus has been on the collaborative development and dissemination of data standards. Key efforts in this area have included development and enhancement of the NPO and ISA-TAB-Nano. ISA-TAB-Nano is currently used by NCI, the NBI Knowledgebase (http://nbi.oregonstate.edu/), and the EU NanoSafety Cluster (http://www.nanosafetycluster.eu/) to enable interoperability between databases. Most recently, the Nano WG established a subgroup focused on developing guidelines for data curation, and is in the process of writing a series of consensus papers on curation workflows, data completeness and quality, curator responsibilities, metadata, and integration between datasets and databases, as an overview of current curation practices and recommendations (Nanomaterial Data Curation Initiative, https://nciphub.org/groups/nanotechnologydatacurationinterestgroup) [20–21].

In line with the goals of this subgroup, the journal Nature Nanotechnology recently published an editorial to announce their plans to participate in Nature’s initiative to improve consistency and reporting of data in life sciences articles [22]. Starting in January 2015, the journal requires the submission of a checklist that ensures authors disclose all the information necessary for others to reproduce their work. This full disclosure includes the deposition of data into comprehensive public databases such as caNanoLab and the Nanomaterial Registry (https://www.nanomaterialregistry.org/). The journal expressed interest in working with communities to develop customized checklists appropriate for specific research fields to streamline data reporting and deposition during the manuscript submission process. As part of this effort, caNanoLab is listed as a recommended data repository for Scientific Data, a Nature journal that publishes descriptions of scientific datasets, and the caNanoLab team participates in the NCIP Nano WG’s Nanomaterial Data Curation Initiative. Increased interactions between caNanoLab and journal publishers are also underway to facilitate the development of reporting guidelines in an effort to increase data deposition at the manuscript submission stage [12].

Federal members of the caNanoLab team participate in the NNI Signature Initiative on Nanotechnology Knowledge Infrastructure (NKI) – enabling national leadership in sustainable design [23]. The purpose of the NNI Signature Initiatives is to rapidly advance science and technology by coordinating the programmatic efforts of member federal agencies in areas identified to be of national importance such as nanotechnology data management. The NKI is focused on major thrust areas, including the creation of a data infrastructure to support data sharing, and management to enable novel nanotechnology-based innovations across disciplines. As such, the NKI works with varied groups to accomplish the initiative’s goals of ultimately sustaining new innovation and knowledge discovery in the design and application of nanomaterials in science.

## Conclusion

Access to detailed nanomaterial characterization data is seen as a prominent need to advance cancer nanomedicines to the clinical environment. To aid this process, caNanoLab will continue to evolve as a valuable resource to the biomedical nanotechnology community through portal enhancements and through integration with other community-identified resources. Plans are underway for a caNanoLab 2.1 release, which will include increased usability and performance enhancements, a Google-like search capability, advanced search and query features, pop-up instructions for data submission fields, and enhancements to the MyWorkspace feature. The caNanoLab 2.1 release will be available in late summer 2015. caNanoLab software is open source and available for download from GitHub for local installation (https://github.com/NCIP/cananolab). This code is customizable, and code contributions back to the community via GitHub are strongly encouraged to support further development of caNanoLab. As part of the evolution of the portal, the caNanoLab team plans to maintain collaborations with other nanomaterial resources used by the community in support of nanomaterial data standards development, integration, and analysis. The future development of caNanoLab will be guided by community practices supporting data interoperability and exchange, such as the use of ISA-TAB-Nano and community developed common web services.

## User Feedback

The caNanoLab team is interested in feedback from the user community on the new caNanoLab features and plans for future enhancements. A discussion forum was created to receive this feedback at https://nciphub.org/groups/cananolab_usability. The team is especially interested in the community’s ideas for needed features, as well as data.
